# P-897. Inconsistent Participant Demographic Reporting in Published Antibiotic Clinical Trials Targeting Multidrug-Resistant Gram-Negative Organisms

**DOI:** 10.1093/ofid/ofae631.1088

**Published:** 2025-01-29

**Authors:** Evangeline Green, Sarah Uhm, Elizabeth Lyden, Sammy Muhia, Samantha Moreno, Cindy Schmidt, Bryan T Alexander, Zanthia Wiley, Jacinda C Abdul-Mutakabbir, Geneva M Wilson, Charlesnika T Evans, Jasmine R Marcelin

**Affiliations:** University of Nebraska Medical Center, Omaha, Nebraska; University of Nebraska Medical Center, Omaha, Nebraska; University of Nebraska Medical Center, Omaha, Nebraska; Nebraska Medicine, Omaha, Nebraska; Western Michigan University, Kalamazoo, Michigan; University of Nebraska Medical Center, Omaha, Nebraska; Nebraska Medicine, Omaha, Nebraska; Emory University, Atlanta, GA; University of California San Diego, San Diego, CA; Hines VA, Hines, Illinois; Northwestern University, Chicago, IL; University of Nebraska Medical Center, Omaha, Nebraska

## Abstract

**Background:**

NIH policies and reporting requirements relating to the inclusion of women and minoritized individuals in clinical trials have evolved since 2000. Over time, prioritization of gender equity in participant recruiting has been successful. However, many studies have demonstrated underrepresentation of participants from minoritized populations. With growing evidence of racialized disparities in antimicrobial resistance and prescribing, clinical trials for new antimicrobials should prioritize equitable participation. We aimed to assess participant demographic reporting in published clinical trials targeting multi-drug resistant gram-negative organisms (MDRGNOs).

Record identification, screening, and inclusion

**Figure:**
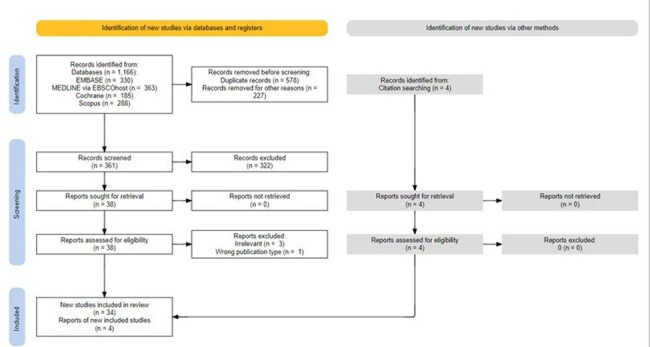

Flow chart for record identification and review leading to the 38 included studies.

**Methods:**

Four databases were searched to identify randomized controlled clinical trials of antibiotic regimens used to treat infections with MDRGNOs published 2010-2023 (Fig1). Participant demographics were extracted from eligible studies and data summarized with descriptive statistics. Fisher’s exact test evaluated associations between variables and Cochran-Armitage tested for trends. P< 0.05 was considered statistically significant.

Study Characteristics

**Figure:**
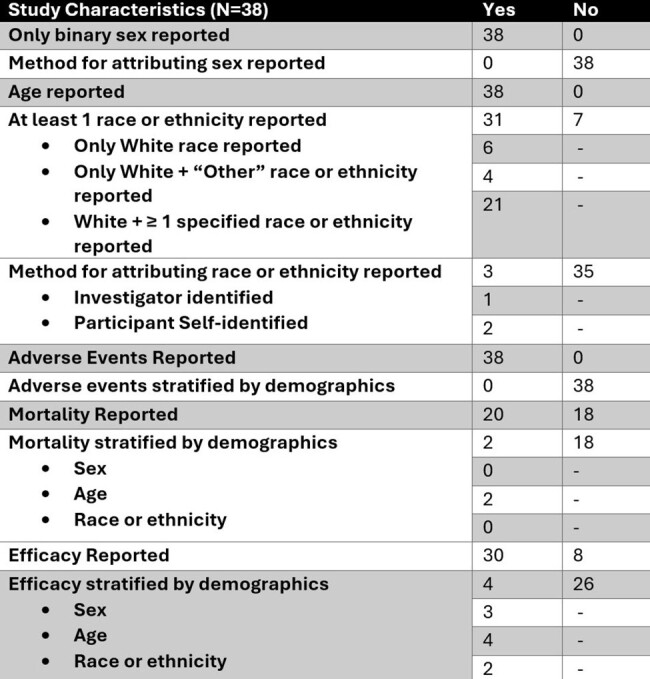

Table showing the characteristics of information reporting across the 38 included studies.

**Results:**

38 full-text articles were included. All studies reported age and binary sex. 31/38 reported race or ethnicity; all 31 reported White race and 10/31 only reported White or Other race (Table). Adverse events, mortality, and efficacy were stratified by demographics 6 times in 5 unique articles. There was no significant pattern in reporting race or ethnicity in published trials for antibiotics targeting MDRGNOs between 2010 and 2023 (Fig2). Among federally funded studies 50% (3/6) reported race or ethnicity compared to 87% (28/32) of industry funded studies, which was marginally significant (p=0.063, Fig3).

**Figure:**
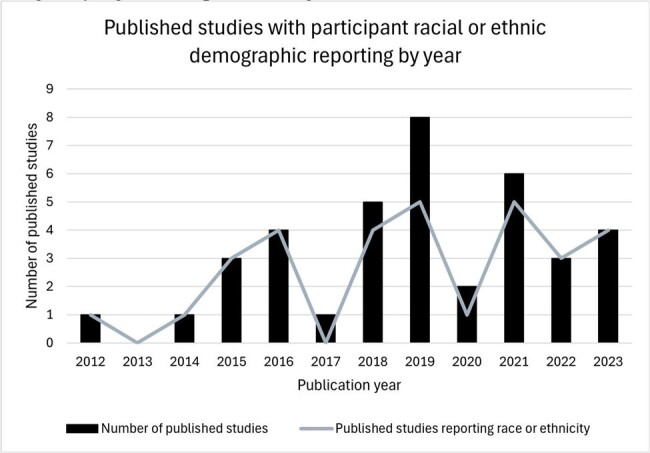

Figure 2 shows the number of published studies reporting race or ethnicity per year (gray line) compared with the number of included studies by year (black bar).

**Conclusion:**

Sex and age reporting occurred across all published clinical trials reviewed focused on treating MDRGNOs. Major study outcomes were rarely stratified by participant demographics. We identified inconsistent reporting of race, ethnicity, and other demographic data. Attribution of demographic data is unstandardized and often not identified. Inconsistent and incomplete reporting limits the ability to accurately study participant demographic representation over time and across studies.

**Figure:**
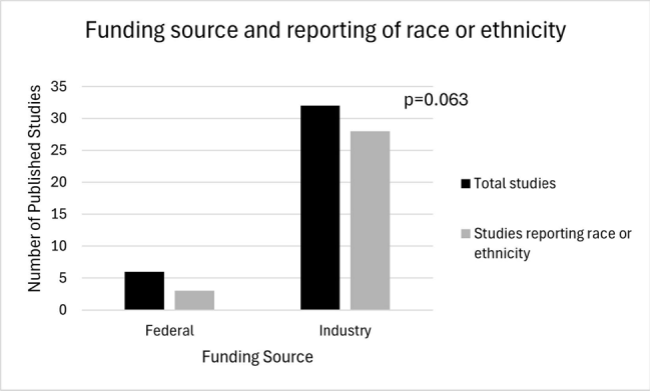

Figure 3 shows the comparison of reported funding source (black bar) with studies reporting race or ethnicity (gray bar). 50% of federally funded studies reported race or ethnicity and 87% of industry funded studies included race or ethnicity.

**Disclosures:**

**Jacinda C. Abdul-Mutakabbir, PharmD, MPH**, CSL Sequiris: Advisor/Consultant|CSL Sequiris: Honoraria|GSK: Advisor/Consultant|GSK: Honoraria|Shionogi: Advisor/Consultant|Shionogi: Honoraria

